# USP1 inhibition suppresses the progression of osteosarcoma via destabilizing TAZ

**DOI:** 10.7150/ijbs.65428

**Published:** 2022-05-01

**Authors:** Putao Yuan, Zhenhua Feng, Hai Huang, Gangliang Wang, Zhijun Chen, Guang Xu, Ziang Xie, Zhiwei Jie, Xiangde Zhao, Qingliang Ma, Shiyu Wang, Yang Shen, Yizhen Huang, Ying Han, Huali Ye, Jiying Wang, Peihua Shi, Xuewu Sun

**Affiliations:** 1 Department of Orthopaedic Surgery, Sir Run Run Shaw Hospital, Zhejiang University School of Medicine.; 2 Key Laboratory of Musculoskeletal System Degeneration and Regeneration Translational Research of Zhejiang Province.; 3 Key Laboratory of Biotherapy of Zhejiang Province, Hangzhou, China.; 4 903RD Hospital of Pla, Hangzhou, China.

**Keywords:** Osteosarcoma, USP1, TAZ, Hippo, Ubiquitin, ML323

## Abstract

Mutations and altered expression of deubiquitinating enzymes (DUBs) profoundly influence tumor progression. Ubiquitin-specific protease 1 (USP1) is a well-characterized human DUB reportedly overexpressed in and associated with maintaining the mesenchymal stem cell status of osteosarcoma (OS); however, the potential mechanisms of USP1 in OS remain poorly understood. In this study, we identified that USP1 directly interacts with Transcriptional Co-Activator With PDZ-Binding Motif (TAZ) in OS cell lines, and with mechanistic analysis indicating that the anti-OS effects of USP1 inhibition could be partially attributed to TAZ instability, with its reduced nuclear accumulation responsible for a subsequent decrease in the expression of downstream genes associated with the Hippo signaling pathway. Moreover, pharmacological inhibition USP1 by ML323 presented the similar effects on Hippo signaling pathway and suppressed OS growth and metastasis both *in vitro* and *in vivo*. Taken together, our results revealed a novel molecular mechanism underlying the function of USP1 in OS and a potential role of ML323 as a therapeutic strategy for the clinical treatment of OS.

## Introduction

Osteosarcoma (OS) is among the most common primary malignant bone tumors that develop in adolescents 1. Despite increases in the 5-year overall survival rates for adolescents with localized disease to 60% to 70% due to supplementation of chemotherapy with surgical resection, clinical outcomes in young patients with OS have not changed substantially over the previous 30 years 2. Moreover, there are numerous problems with traditional treatment methods, with a large number of patients prone to developing chemoresistance 3. Therefore, there remains an ongoing need to further understand the molecular mechanisms underlying OS oncogenesis and explore novel methods to address therapeutic limitations.

Ubiquitination and deubiquitination are dynamic and reversible processes, with deubiquitination involved in removal of the ubiquitin chain or ubiquitin from substrate proteins 4. This process is specifically catalyzed by a large class of proteins referred to as deubiquitinases (DUBs) 4. To date, there exist ~100 human DUBs 5, 6, with related genetic deficiencies and functional dysregulation profoundly influencing numerous human diseases 7, ranging from neurological disorders to various types of cancer 8, 9. Recent studies have reported the roles of DUBs in OS. Ubiquitin C-terminal hydrolase L1 (UCHL1) mediates the AKT signaling pathway to accelerate OS progression 10, BRCA1-associated protein 1 (BAP1) suppresses the proliferation and migration capacities of OS cells by inhibiting phosphoinositide 3-kinase/AKT signaling 11. Additionally, ubiquitin-specific peptidase 7 (USP7) induces epithelial-mesenchymal transition (EMT) in OS by activating Wnt/β-catenin signaling pathway 12. These findings suggest DUBs as potential diagnostic and therapeutic targets in OS treatment.

USP1 is member of the largest family of deubiquitinating enzymes (USPs) and among the best-characterized human DUBs. USP1 is involved in deubiquitination of proliferating cell nuclear antigen (PCNA), Fanconi anemia group D2 (FANCD2) and group I (FANCI), so as to be considered a vital regulatory factor in the process of DNA damage and repair response 13-15. Moreover, USP1 maintains mesenchymal stem cell status in OS by deubiquitinating inhibitors of DNA binding (IDs) 16. In U2OS cells (a human OS cell line), silencing USP1 attenuated U2OS proliferation and invasion 17. However, to our knowledge, the specific role and underlying mechanisms of USP1 in OS progression have not been fully clarified. There remains an urgent need to further explored its molecular mechanisms in mediating OS oncogenesis. In addition, as a selective inhibitor of USP1, ML323 exerted anti-metastatic effects in breast cancer 18 along with the abilities to sensitize resistant OS cells to cisplatin 19. Given the regulatory effects of USP1 in U2OS cell lines, further explore the role of ML323 in osteosarcoma may address the current therapeutic limitations and reveal new strategies for the clinical treatment of OS.

In this study, we identified that USP1 directly interacts with TAZ in OS cells. And the anti-OS properties presented with USP1 deletion can be partially attributed to the promoted TAZ poly-ubiquitylation via K11 and K29 linkage for its subsequent proteasomal degradation, which resulted in dysfunction of the Hippo signaling pathway. Overexpression of TAZ partially abrogated the antitumor effects of USP1 deletion in OS cell lines. Additionally, pharmacological inhibition of USP1 by ML323 presented the similar effects on Hippo signaling pathway and suppressed OS progression both *in vitro* and *in vivo*. Taken together, our results revealed a novel molecular mechanism underlying the functions of USP1 in OS and a potential role for ML323 as a therapeutic strategy for the clinical treatment of OS.

## Material and methods

### Ethics

Laboratory Animal Care and Use Guidelines, which is formulated by the National Institutes of Health and authorized by the Ethics Committee of Sir Run Run Shaw Hospital, were carried out strictly in animal experiments. Experiments including the feeding, treatment along with euthanasia of animals were rigorously adhered to the specific guidelines from panel.

### Clinical samples

The chondroma and osteosarcoma samples were obtained from surgical patients without neoadjuvant chemotherapy. Bone tissues were derived from patients with hip replacement. All the patients signed informed consent and authorized the usage of specific specimens for the appropriate research. Pathology department of Sir Run Run Shaw Hospital took part in identifying the characteristics of chondroma and osteosarcoma specimens on basis of the standards established by World Health Organization. Surgically excised specimens were preserved at -80 °C for subsequent RNA extraction or fixed by formalin followed with paraffin embedding, and then used for further experiments, including immunohistochemical staining.

### Reagents and antibodies

The compound ML323 was purchased from Medchem Express (USA), and preserved in -20 for further utilizing. Dimethylsulphoxide (DMSO) was purchased from Sigma (Sigma-Aldrich, St. Louis, MO, USA). Dulbecco's Modified Eagle Medium (DMEM), fetal bovine serum (FBS) along with phosphate-buffered saline (PBS) were purchased from Gibco Life Technologies. Cell counting kit-8 (CCK-8) was purchased from Dojindo Molecular Technology. Specific antibodies against PCNA, Caspase 3, cyclin D1, Vimentin, MMP13, TEAD, Runx2, C-Myc, were purchased from Cell Signaling Technology (Boston, USA), antibodies against USP1 were purchased from Biorbyt (Cambridge, UK), antibodies against TAZ (Transcriptional Co-Activator with PDZ-Binding Motif) were purchased from Santa Cruz Biotechnology (Santa Cruz, USA) and antibodies against E-Cadherin, N-cadherin, CYR61 were purchased from Proteintech (Beijing, China). MG132 and Chloroquine (CQ) were purchased from MedChem Express (New Jersey, USA). All other chemicals used were of analytical grade and indicated in the article.

### Cell culture

The human osteoblast cell line hFOB1.19 along with the human osteosarcoma cell lines including 143B, HOS, U2OS, MG63 and SAOS were purchased from American Type Culture Collection (ATCC). All cell lines were confirmed to be mycoplasma-free by Venor GeM Mycoplasma Detection Kit (Minerva Biolabs, Berlin, Germany) before cell experiment. All cells were cultured in Dulbecco's modified Eagle's medium (DMEM) containing 1% penicillin/streptomycin and 10% fetal bovine serum (FBS). The 293T cell line and osteosarcoma cells were incubated in a 37 °C cell incubator with 5% CO_2_. The cell culture conditions of hFOB1.19 were similar to those of osteosarcoma cells, with the exception of temperature (34 °C).

### CCK-8 assay

CCK8 assay was performed to detect the effect of ML323 on the cell viability of 143B and HOS cells, so as to determine the appropriate experimental concentration of ML323 and its impacts on proliferation of osteosarcoma cells. In brief, 143B and HOS cells were incubated in 96-well plates with a density of 4 × 10^3^ cells per well. Then, cells were exposed to different concentrations of ML323 (0-64 μmol/L) for 24, 48 or 96 hours. At the corresponding time point mentioned above, the medium of each well were replaced with 100 μL fresh DMEM containing 10% CCK8 reagent (Sigma-Aldrich, St. Louis, MO, USA) and then the absorbance value of each well was measured by ELX800 absorbance microplate reader (BioTek Instruments, Vermont, USA) at 450 nm.

### Colony-formation assay

For colony-formation assay, 143B and HOS cells were seeded in 6-well plates at a density of 1× 10^3^ cells per well. Then the cells were treated with different concentrations of ML323 (0, 8, 16 32 μmol/L) after they were completely adherent. After about 7-14 days of culture, colonies were fixed with 4% paraformaldehyde for 20 min, and then stained with 1% crystal violet for another 20 min at room temperature. The number of colonies was then counted and analyzed after washing by PBS.

### Soft agar colony formation assay

143B and HOS cells were inoculated in semisolid agar medium (0.5% agarose/PBS culture medium with a 0.6% agarose/PBS bottom layer in a 6-well plate) at a density of 2.5×10^4^ cells/well. After 24 hours, cells were treated with ML323 at different concentrations (0, 8, 16 or 32 μmol/L). Representative images of cell colonies were then photographed by an inverted microscope (Nikon, Tokyo, Japan) on 0, 5 and 10 days, respectively.

### EdU assay

Firstly, 143B and HOS cells were seeded in 6-well plates with a suitable density. After adherence for 24 hours, the cells were starved for about 8 hours with FBS-free DMEM to synchronize cell cycle of each wells. Then cells were exposed to 0, 8, 16 or 32 μmol/L ML323 for 48h. After that, the medium of each well were replaced with fresh DMEM containing 10 μM EdU and incubated at 37 °C, with 5% CO_2_ for another 2 hours. Then fixed with 4% paraformaldehyde for 15 min and incubated with 0.3% Triton-X-100 for 15 min. Afterwards, the fixed cells were incubated with Click reaction buffer for 30 min away from light. Subsequently, the nuclei were stained with Hoechst dye 33342. Cellular proliferation rate was calculated in terms of the manufacturer's instructions (BeyoClick™EdU-488 Cell Proliferation Detection Kit, Beyotime Biotechnology, Shanghai, China).

### Flow cytometry

143B and HOS cells were inoculated into 6-well plates with a suitable density. Then the cells were exposed to different concentrations of ML323 (0, 8, 16 32 μmol/L) for 48h after they were completely adherent. For cell apoptosis analysis, adherent cells were harvested along with the cells in the supernatant. The obtained cells were incubated with FITC-conjugated Annexin V and PI, CA, USA in dark for about 15 minutes after washed twice by PBS. Finally, the samples were analyzed using the Accuri C6 (BD Biosciences, SanDiego, USA). For cell cycle assay, cells need an extra 8 hours of starvation before the treatment with different concentrations of ML323 to synchronize cell cycle of each wells. Then, the cells were digested, centrifuged, and washed by PBS. Subsequently, mixed each sample with 1 ml DNA Staining solution, 10 μl Permeabilization solution (Cell cycle staining Kit, MultiSciences, Hangzhou, Chian), and incubated for 30 minutes at room temperature away from light. Finally, the distribution of cells in different cycles was detected by Accuri C6 (BD Biosciences, SanDiego, USA) as well.

### Wound-healing assay

143B and HOS cells were seeded into Six-well plates and then scratched by a 200-μL pipette tip to form two perpendicular wounds per well when the cells were grown to about 80% confluency. Subsequently, the wells were washed twice with PBS to wash off the un-adherent cells and treated with 0, 8, 16 or 32 μmol/L of ML323. The images of wounds from the same position were photographed by an inverted microscope (Nikon, Tokyo, Japan) at 0 and 24H, respectively. Finally, ImageJ software (NIH, Bethesda, MD, USA) was utilized to quantify the ratio of the area of wound healing within 24 hours.

### Transwell migration and invasion assays

12-well Transwell chambers were utilized to evaluate invasion and migration capability of 143B and HOS cells. For migration assays, cells were re-suspended with FBS-DMEM and seeded into upper chambers with a density of 5× 10^4^ cells/well. The lower chambers were added 500 µL DMEM containing 10% FBS. After 24- hours-incubation with different concentrations of ML323 (0, 8, 16 32 μmol/L), part of cells will migrate from the upper to the lower side of chamber. Then chambers were washed with PBS and fixed with 4% paraformaldehyde for 20 min. Cells were then stained with 1% crystal violet for about 15 min. Afterwards, wash the chambers by PBS for five times and wiped off the cells which were detained in the upper side. Finally, images were captured by microscopy. For invasion assay, the general process was similar to migration assay except that chambers for the invasion assay were pre-coated with Matrigel basement membrane matrix according to the manufacturer's instructions (BD Science, Bedford, MA, USA).

### Quantitative real-time PCR (qRT-PCR)

Total RNA was extracted from 143B and HOS cells treated with different concentrations of ML323 (0, 8, 16 32 μmol/L) for 48h according to the instructions of RNeasy kit (Invitrogen, California, USA). Then the extracted mRNAs were subjected to reverse transcription to cDNAs with kits from Accurate Biotechnology (Hunan, China). Obtained cDNAs were quantified with SYBR® Green Premix Pro Taq HS qPCR kit (Accurate Biotechnology, Hunan, China), and detected by an ABI Prism 7500 system (Applied Biosystems, Massachusetts, USA). The 2^-ΔΔCt^ method was then utilized to figure up the relative expression of target genes at transcriptional level. The concentrations of specific cDNAs were normalized to ACTB. Each experiment were repeated three times independently. All primers are listed in Table 1.

### Western blotting analysis

143B and HOS cells were seeded into Six-well plates at a density of 5 × 10^4^ cells/well. Cells were then exposed to different treatments for 48h after complete adherence. Radioimmunoprecipitation assay (RIPA) lysis buffer (Sigma-Aldrich, St. Louis, MO, USA) containing protease and phosphatase inhibitors was then used to extract the total proteins from each sample. In brief, 150 μL of mixed Ripa lysis buffer were added into each well of the six-well plates, which was then cracked on ice for 30 minutes. The liquid in each well was collected as completely as possible, and centrifuged at 4 °C for 15 minutes under the condition of 14000 g. Then mixed the obtained supernatant with 5× loading buffer in proportion and boiled at 100 °C for 10 min. The obtained protein samples were then stored at -20 °C. For western blotting, proteins were separated by SDS-PAGE (8%-12%) and transferred to 0.45-μm PVDF membranes (Bio-Rad, Hercules, CA, USA). The membranes were then blocked in 5% non-fat dry milk at room temperature for 1 hour, after which the blocked membranes were incubated with specific primary antibodies overnight at 4 °C. On the following day, membranes were washed three times with TBST buffer and incubated at room temperature for 1 h with the corresponding secondary antibody. Followed by another three times washed with TBST buffer, the protein band were visualized by LAS-4000 Science Imaging System (Fujifilm, Tokyo, Japan). The results were analyzed with Image J.

### Cell transfection

Cells were inoculated in cell-culture-plates at the appropriate density. Then, cells were transfected with the specific SiRNA or plasmids using Lipofectamine 3000 and P3000 (Invitrogen, California, USA) in a proper proportion following the manufacturer's instructions. After 8 hours of transfection, the medium was replaced with fresh DMEM containing 10% FBS and cells were cultured for the indicated days for further experiments. USP1-SiRNA and negative control (SiNC) were purchased from Ribo Life Sience (Suzhou, China). Flag-tagged TAZ plasmids in the pEnter vector was purchased from Vigenebio (Shandong, China). HA-tagged ubiquitin, K11, K29, K33, K48, and K63 plasmids in the PRK5 vector were provided by Shao-cong Sun (MD Anderson Cancer Center, Houston, Texas, USA).

### Immunoprecipitation (IP) assay

Firstly, the pretreated cells were lysed by mixed Radioimmunoprecipitation assay (RIPA) lysis buffer (Sigma-Aldrich, St. Louis, USA) as mentioned above, the obtained protein lysate was then immunoprecipitated with the corresponding primary antibody according to the instructions and placed in 4 °C overnight. Subsequently, the obtained antigen-antibody mixture were incubated with 50 μL of protein A/G-agarose (50% v/v) Yeasen, Shanghai, China) at 4 °C for about 8 hours to form protein A/G-agarose-antigen-antibody complexes. Finally, the magnetic beads in the complex were removed by thermal denaturation to obtain the binding proteins we needed. Binding proteins were then resolved by SDS-PAGE for further western blotting.

### Immunofluorescence staining

Firstly, the processed cells were washed three times with PBS and fixed in 4% paraformaldehyde for 20 mins. Then, 0.5% Triton X-100 was utilized to permeabilized the fixed cells for 30 mins before an 1-hour- blockade with 5% bovine serum albumin (BSA) (Fudebio, Hangzhou, China). After which, cells were incubated with specific primary antibodies overnight at 4 °C. The corresponding secondary antibodies (Invitrogen, USA) were added to the cells the next day. Finally, cells were washed with PBS for three times and stained with DAPI (Beyotime Institute of Biotechnology, Shanghai, China) for 10 mins to dye the nucleus. Image of immunofluorescence were further acquired from a fluorescence microscope (Olympus, Tokyo, Japan).

### Drug Affinity Responsive Target Stability Assay (DARTS)

Mammalian Protein Extraction Reagent (M-PER) (Thermo Fisher Scientific, Waltham, Massachusetts, USA) containing protease and phosphatase inhibitors was used to lyse the cells from each sample. TNC buffer (50 mM Tris-HCl PH=8.0, 50 mM NaCl and 10 mM CaCl_2_) were then added to the cell lysates and mix well. The mixed lysates were then added with vehicle (DMSO) or ML323 and incubated at room temperature for 1 hour. Then, the prepared lysates were digested at room temperature by Pronase (Roche, Basel, Switzerland) for about 15 minutes and resolved by SDS-PAGE for further western blotting.

### Subcutaneous xenograft tumorigenesis model

Nude mice (male, 6 weeks old) were purchased from Shanghai SLAC Laboratory Animal Co and used to establish xenograft tumorigenesis model. Briefly, each nude mouse was injected subcutaneously into both sides of flank with 100 μl cell suspension containing 5×10^6^ 143B stable cells. Mice that had developed primary tumors were randomly divided into three groups by day 3, and injected intraperitoneally with ML323 (5 mg/kg or 10 mg/kg) or vehicle solution (Control) once in two days. Tumor diameter and body weight of mice were measured every two days before each injection. The nude mice were sacrificed three weeks after intraperitoneal injection, the tumors and vital organs of mice in each group were harvested. Part of the tumors were utilized to extract proteins for further western blotting, while the rest were preserved along with the harvested vital organs and fixed with 4% paraformaldehyde for subsequent experiments.

### Orthotopic xenograft tumorigenesis model

Nude mice (male, 6 weeks old) were purchased from Shanghai SLAC Laboratory Animal Co and used to establish orthotopic xenograft tumorigenesis model. Briefly, each nude mouse was injected into the cavity of the tibia with 100 μl cell suspension containing 5×10^6^ 143B stable cells. Mice that had developed primary tumors were randomly divided into three groups by day 3, and injected intraperitoneally with ML323 (5 mg/kg or 10 mg/kg) or vehicle solution (Control) once in two days. The nude mice were euthanized three weeks after intraperitoneal injection, the tibia of orthotopic xenograft model from each group were harvested for micro CT scanning.

### Tail vein metastasis model

Briefly, Luminescence-labeled 143B cell lines were suspended in sterile PBS to a suitable concentration. Nude mice (male, 6 weeks old) were then injected with 1 × 10^6^ prepared stable cells via the tail vein and randomly divided into three groups, injected intraperitoneally with ML323 (5 mg/kg or 10 mg/kg) or vehicle solution (Control) once in two days. Subsequently, an *in vivo* bioluminescence imaging system (Xenogen, Caliper Life Sciences, Waltham, USA) was utilized to imaged lung metastasis on day 7, day 14 and day 21, respectively. Acquired images were then analyzed by Living Image 4.1 software (Xenogen, Caliper Life Sciences, Waltham, USA). Lungs from each nude mouse were fixed with 4% paraformaldehyde for the subsequent H&E staining. Three nonadjacent levels of each lung specimens were then selected for lung metastasis counting and each consolidation foci were counted as a lung metastasis. The average of three levels was subsequently calculated as the number of tumor metastasis in the corresponding lung tissue.

### Histology analysis and immunohistochemistry

The normal bone, chondroma, osteosarcoma samples along with tumors, vital organs, tibia and lungs from tumorigenesis models were preserved as mentioned above. The specimens were then subjected to section into a thickness of about 5μm and stained with haematoxylin and eosin (H&E) for the subsequent histology analysis. As for immunohistochemistry, sections were stained using a histo-stain SABC kit (CWBIO, Shanghai, China) according to the instructions of manufacturer. The specific primary antibody was used at a dilution of 1:100 in this study. Image-Pro Plus 6.0 (NIH, Bethesda, MD, USA) was then used to calculate the percentage of positive cells, which is determined by the proportion of cells positive for the marker in all cells. All histological analyses were independently reviewed in parallel by specialists from the pathology department of Sir Sun Shaw Hospital.

### Statistical analysis

GraphPad Prism 5.0 (GraphPad Software, La Jolla, CA, USA) was carried to perform data analyses. All quantitative data are presented as mean ± SD. Statistical significance was analyzed with the unpaired, two-tailed Student's t test, unless noted otherwise. P values less than 0.05 was considered to be statistically significant. Significance level was presented as either *P < 0.05, **P <0.01 or ***P < 0.001.

## Results

### USP1 directly interacts with TAZ in OS cells

**Figure 1:**
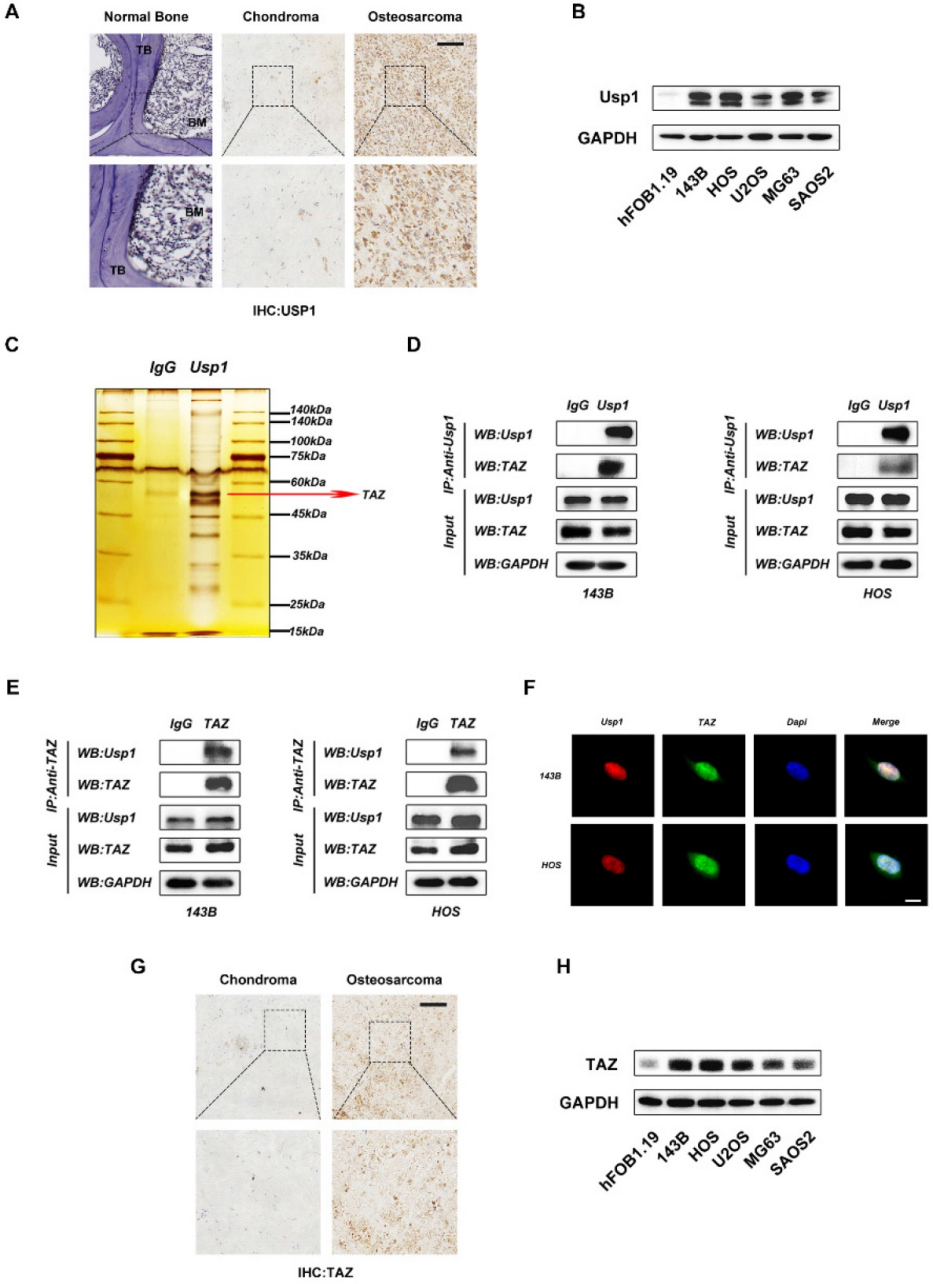
** USP1 directly interacts with TAZ in OS cells. A.** Histological analysis of osteosarcoma tissues compared with normal bone and chondroma. The expression of USP1 was then identified by immunohistochemistry staining. **B.** The expression of USP1 in hFOB1.19 cells(a osteoblast cell line of human origin) and several human osteosarcoma cell lines, including 143B, HOS, U2OS, MG63 and SAOS2 cells, were detected by Western blot. **C. D. E.** Co-IP assay was performed to determine the connection between USP1 and TAZ. The silver staining results presented that one of the differential bands compare to control immunoglobulin G (IgG) is about 55 kdA which is similar with TAZ (C). Further Western blot assay confirmed the interaction between USP1 and TAZ in both 143B and HOS cells (D and E). **F.** USP1 and TAZ are localized in the nucleus of OS cells, as examined by immunofluorescence staining. DAPI was used to stained the nuclei. **G. H.** The expression level of TAZ was relatively higher in both osteosarcoma tissues and cell lines by comparing with chondroma sections and hFOB1.19 cells (an osteoblast cell line of human origin), as presented in immunohistochemistry assay (G) and Western blot assay (H). Data represents the means ± SD. The images and data presented were acquired from and represented three independent experiments. P*< 0.05, P** < 0.01, P*** < 0.001 in comparison with the control group.

USP1 is reportedly overexpressed in some tumor tissues, including gastric cancer, breast cancer and lung cancer. To further verify this in OS, we detected the expression of USP1 in OS tissues relative to levels in normal bone and chondroma by immunohistochemical. The results revealed USP1 overexpression in OS tissues (Fig. 1A). Moreover, by comparing with hFOB1.19 cells (a human osteoblast cell line), we found that USP1 expression was elevated in several OS cell lines as well, with particularly high expression observed in 143B and HOS cells (Fig. 1B). Therefore, we selected 143B and HOS cells for subsequent experiments. To further investigate the underlying mechanisms of USP1 in OS, we performed co-immunoprecipitation (Co-IP) assays. Compared with controls [immunoglobulin G (IgG) in 143B cells], silver staining results showed enrichment of several bands of proteins precipitated by an antibody against USP1, with one of these at ~55 kDa (Fig. 1C). Given that TAZ, an OS oncogene about 55 kDa, was reported to form a complex with USP1 to affect the metastatic properties of breast cancer 21, we speculated whether this association exists in OS and confers to the regulatory effects of USP1. Thus, we performed western blot to identify the Co-IP samples precipitated by the USP1 antibody from OS cells, finding that USP1 directly interacts with TAZ in both 143B and HOS cells (Fig. 1D), with reverse Co-IP experiments further confirming the association (Fig. 1E). Additionally, immunofluorescence verified localization of USP1 and TAZ in the nucleus (Fig. 1F). Subsequent immunohistochemistry along with western blot assay jointly identified elevated TAZ expression in OS tissues and cell lines (Fig. 1G and 1H), which confirmed the consistency between the elevated expression of USP1 and TAZ in OS.

### USP1 depletion promotes the ubiquitination of TAZ and results in dysfunctional Hippo signaling pathway

**Figure 2:**
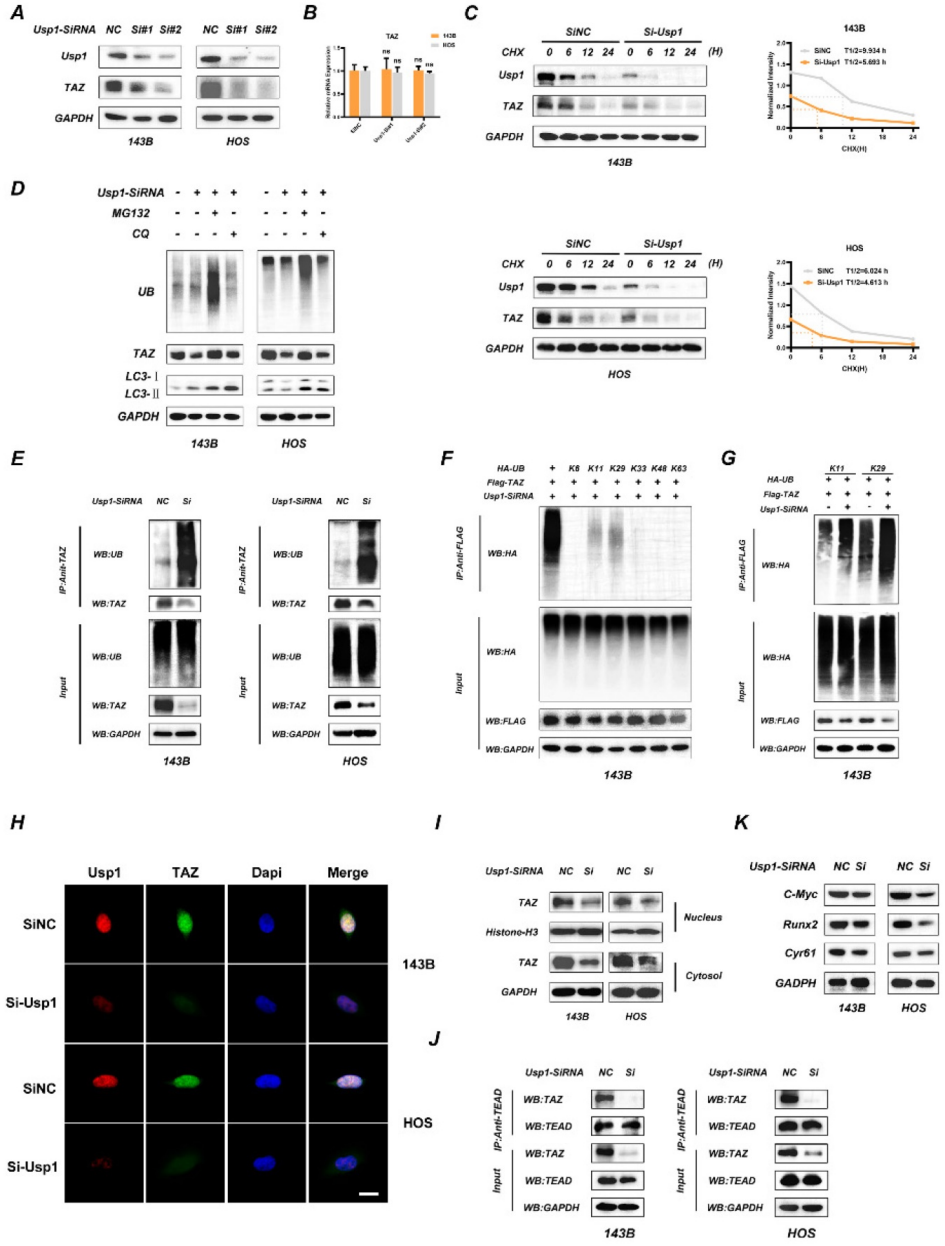
** USP1 depletion promotes the ubiquitination of TAZ and results in dysfunctional Hippo signaling pathway. A, B.** USP1 depletion significantly decreased TAZ protein levels in OS cells, as determined by Western blot (A). While the mRNA of TAZ remained unchanged under USP1 depletion conditions (B). **C.** USP1 depletion promoted the degradation of TAZ. After treating the OS cells with cycloheximide (CHX) at different time points as indicated, the expression of endogenous TAZ protein was analyzed by Western blot. **D.** 143B and HOS cells were transfected with Usp1-SiRNA mixture or negative control (SiNC) for 48h, and then MG132 (20 µM, 6 hours) or chloroquine (CQ) (10 µM, 20 hours) were added as indicated. Endogenous ubiquitin, TAZ and LC3 levels were analyzed by Western blot. **E.** USP1 depletion promoted the ubiquitination of TAZ. 143B and HOS cells were transfected with Usp1-SiRNA mixture or negative control (SiNC) for 48h. Cell lysates was immunoprecipitated with the anti-TAZ antibody and TAZ ubiquitination was then examined by Western blot. **F, G.** USP1 depletion specifically increased the K11and K29-linked ubiquitination of TAZ, as confirmed by Co-IP assays. **H, I.** The accumulation of TAZ in nucleus were reduced in case of USP1 depletion in OS cells, as confirmed by Immunofluorescence (H) and Western Blot assay (I). **J.** OS cells were transfected with Usp1-SiRNA mixture or negative control (SiNC) for 48h, then Co-IP assay was performed to detected the interaction of TAZ and TEAD. **K.** The expression of downstream genes in Hippo signaling pathway was reduced in case of USP1 depletion, as detected by Western Blot. Data represents the means ± SD. The images and data presented were acquired from and represented three independent experiments. P*< 0.05, P** < 0.01, P*** < 0.001 in comparison with the control group.

To further determine the association between USP1 and TAZ, we transfected OS cells with two separate siRNA mixtures against USP1 and found that USP1 depletion significantly decreased TAZ protein levels in OS cells (Fig. 2A), whereas no significant change was observed at mRNA levels according to qRT-PCR (Fig. 2B). Considering the deubiquitination properties of the USP1, we speculated that USP1 modulate TAZ expression through the proteasome pathway. Consistent with the conjecture, USP1 depletion accelerated the degradation of TAZ as proved by cycloheximide (CHX) treatment (Fig. 2C). Moreover, we found that USP1-depletion induced TAZ decline could be partially reversed by proteasome inhibitors MG-132, while was not affected by chloroquine (CQ) (a lysosome inhibitor) (Fig. 2D). We thus performed Co-IP assays to analyze the ubiquitination level of TAZ. The results exhibited that USP1 depletion significantly increased the ubiquitination level of TAZ in OS cells (Fig. 2E). Subsequently, we used 143B cells to further investigate which subtypes of ubiquitin chains participated in TAZ modification and were regulated by USP1. As shown, USP1 depletion specifically increased the K11 and K29-linked ubiquitination, nonclassical ubiquitin chains involved in the degradation of proteins (Fig. 2F and 2G). Considering that TAZ acts as a co-activator and functions by translocating into the nucleus, we performed immunofluorescence assays to evaluate the accumulation of TAZ in nucleus, and found that USP1 depletion reduced the intranuclear cumulants of TAZ (Fig. 2H). The similar results were further supported by western blot assays (Fig. 2I). We then assessed the effect of USP1 depletion on the interaction between TAZ and TEAD transcription factors by Co-IP assays, with the results showing that USP1 depletion effectively suppressed these interactions in OS cells (Fig. 2J). Furthermore, we observed decreased levels of cysteine-rich angiogenic inducer 61 (CYR61), c-Myc, and runt-related transcription factor 2 (RUNX2), downstream components of the Hippo signaling pathway, following USP1 depletion (Fig. 2K). These results support our hypothesis that USP1 regulate the expression of TAZ in OS cells by ubiquitination modification, which then influence the Hippo signaling pathway.

### TAZ overexpression partially reverses the anti-tumor effects of USP1 depletion

**Figure 3:**
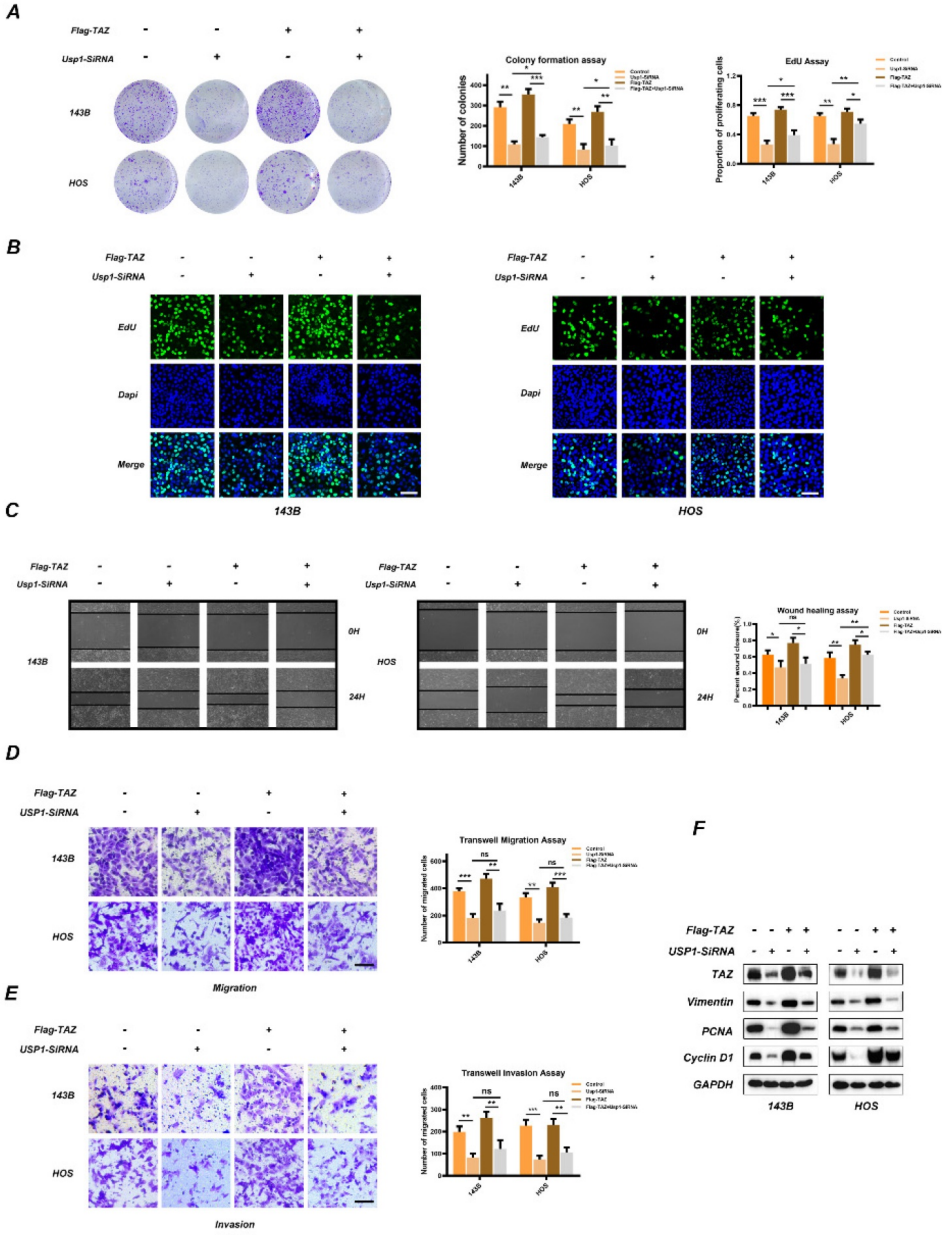
** TAZ overexpression partially reverses the anti-tumor effects of USP1 depletion.** 143B and HOS cells were transfected with USP1-SiRNA mixture, negative control (SiNC), Vector or Flag-TAZ plasmids as indicated. **A, B.** The anti-proliferation properties of USP1 depletion was partially reversed by overexpressing TAZ, as exhibited by Colony formation assays (A) and EdU assays (B) **C.** The migration abilities of OS cells were then evaluated by Wound-healing assay. **D, E.** Transwell migration and invasion assays demonstrated that TAZ overexpression cannot completely reverse the USP1 depletion mediated anti-migration and invasion abilities. **F.** The OS cells were transfected with corresponding SiRNA or plasmids as indicated for 48h, extracts were then utilized for Western blot to determine the expression of related proteins. Data represents the means ± SD. The images and data presented were acquired from and represented three independent experiments. P*< 0.05, P** < 0.01, P*** < 0.001 in comparison with the control group.

To determine whether TAZ is involved in USP1-mediated proliferation, migration, and invasion properties of OS, we transfected Flag-labeled TAZ plasmids into 143B and HOS cells. Colony-formation assays confirmed that overexpression of TAZ partially reversed the antiproliferative role of USP1-depletion in 143B and HOS cells (Fig. 3A), with these results confirmed by EdU assay (Fig. 3B) as well. Besides, wound-healing assays revealed that USP1-deletion slowed the migration of osteosarcoma cells, while overexpression of TAZ merely reversed this performance in HOS cells (Fig. 3C). Moreover, in the Transwell migration and invasion assays, the effects indeed caused by USP1-deletion cannot be completely rescued by overexpressing TAZ (Fig. 3D and 3E). Subsequent western blot analysis indicated that TAZ overexpression partially reverse levels of PCNA and Cyclin D1 which are correlation with cell proliferation while vimentin was not affected (Fig. 3F). These findings suggested that TAZ partially accounts for the anti-proliferation effect of USP1 depletion.

### Inhibition of USP1 by ML323 suppressed proliferation, migration and invasion of OS cell lines

**Figure 4:**
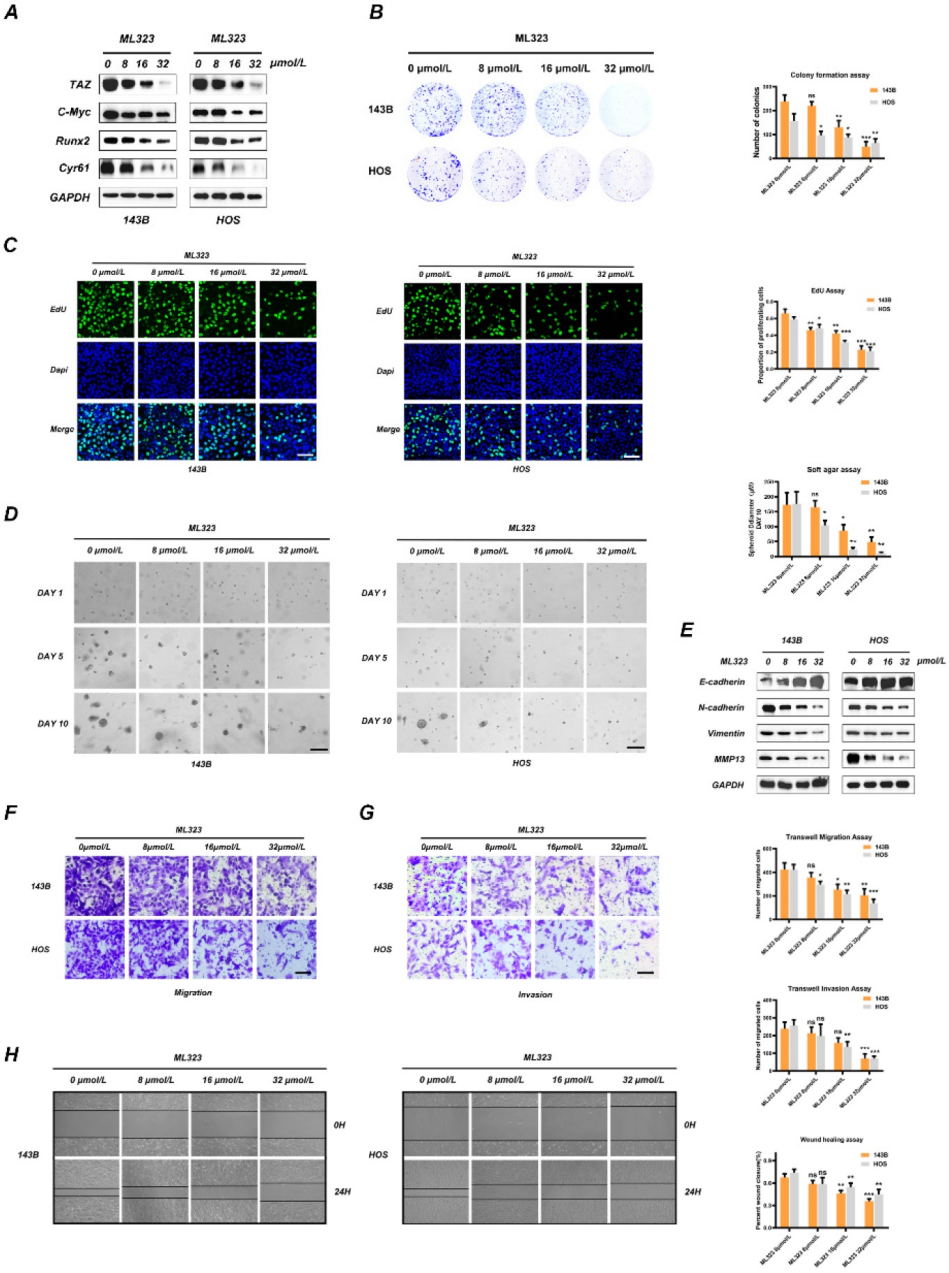
** Inhibition of USP1 by ML323 suppresses proliferation, migration and invasion of OS cell lines. A.** The expression of TAZ and downstream genes in Hippo signaling pathway was reduced with the stimulation of ML323 for 48h, as detected by Western Blot. **B.** The ability of OS cells in forming colonies was weakened after exposing to different dosage of ML323, as determined by colony formation assay. **C.** Functions of ML323 in osteosarcoma cells proliferation as evaluated by EdU assay. DAPI was utilized to stain the nuclei. Cells in S phase can be stained by both EdU and DAPI. **D.** 143B and HOS cells were subjected to different dosage of ML323, and then utilized Soft agar colony formation assay to assessed their ability of proliferation. Images were acquired at 1, 5 and 10 days, respectively. **E.** The expression levels of EMT phenotype-related genes were measured by Western Blot assay after the stimulation with different dosage of ML323 range from 0 to 32 µmol/L as indicated for 48h. **F, G.** The migration and invasion capacities of 143B cells and HOS cells with the treatment of ML323 were evaluated by transwell migration and invasion assays. **H.** The wound-healing assay presented that ML323 suppressed migration capacity of 143B and HOS cells in a concentration relative manner. Images were acquired at 0 and 24h respectively for each group. Data represents the means ± SD. The images and data presented were acquired from and represented three independent experiments. P*< 0.05, P** < 0.01, P*** < 0.001 in comparison with the control group.

As a selective inhibitor of USP1, ML323 has been confirmed to sensitizes resistant OS cells to cisplatin, while its role in the progression of OS remains poorly understood. To clarify the effect of ML323 in OS, we first evaluated its cytotoxicity in 143B and HOS cells at 24/48/96h with a Cell Counting Kit-8 (CCK-8) assay, finding half-maximal inhibitory concentration (IC50) values for ML323 at 48 h of 42.41 μmol/L and 40.89 μmol/L in 143B and HOS cells, respectively (Fig. S3B). Therefore, we selected 32 μmol/L as the maximum experimental concentration. Subsequently, DARTS assays were performed to identified the interaction between ML323 and USP1 in OS cells. The results presented that USP1 was resistant to the degradation effect of pronase in case of ML323 treatment, which identified USP1 as the pharmacological target of the ML323 (Fig S3C). In addition, ML323 treatment reduced the expression of TAZ, C-Myc, Runx2 and CYR61 in OS cells, similar to the results of USP1 depletion (Fig 4A). We then evaluated the effect of different ML323 concentrations (0, 8, 16, or 32 μmol/L) on OS cell proliferation by colony-formation assays (Fig. 4B). The results of colonies from each group indicated that ML323 suppressed the proliferation of OS cells. Similar results were obtained from 5-ethynyl-2'-deoxyuridine (EdU)-incorporation assays and soft agar colony-formation assays (Fig. 4D and 4E). However, ML323 has no obvious effects on the apoptosis levels of OS cells, as confirmed by flow cytometric analysis and western blot (Fig S3D and S3E). EMT is an evolutionarily conserved reversible biological process aberrantly activated in various cancers to promote a transformation from an epithelial to a mesenchymal phenotype. Therefore, we detected expression changes of genes involved in EMT by western blot and immunofluorescence assay. The results demonstrated that ML323 downregulated levels of N-cadherin, vimentin, and matrix metalloproteinase 13 (MMP13), while the content of E-cadherin was upregulated (Fig. 4E and S3F). Additionally, EMT-related genes presented the same changes at mRNA levels as confirmed by qRT-PCR (Fig. S3G). Then, the alterations in migration and invasion capacity were further assessed by transwell migration and invasion assays along with wound-healing assays. The results were consistent between three assays and revealed that the migratory and invasive abilities of the OS cells were predominantly abrogated in the presence of ML323 (Fig. 4F, 4G and 4H). Taken together, these findings revealed that inhibition of USP1 by ML323 inhibits proliferation, migration, and invasion in OS cell lines.

### USP1 inhibition by ML323 suppresses OS progression *in vivo*

**Figure 5:**
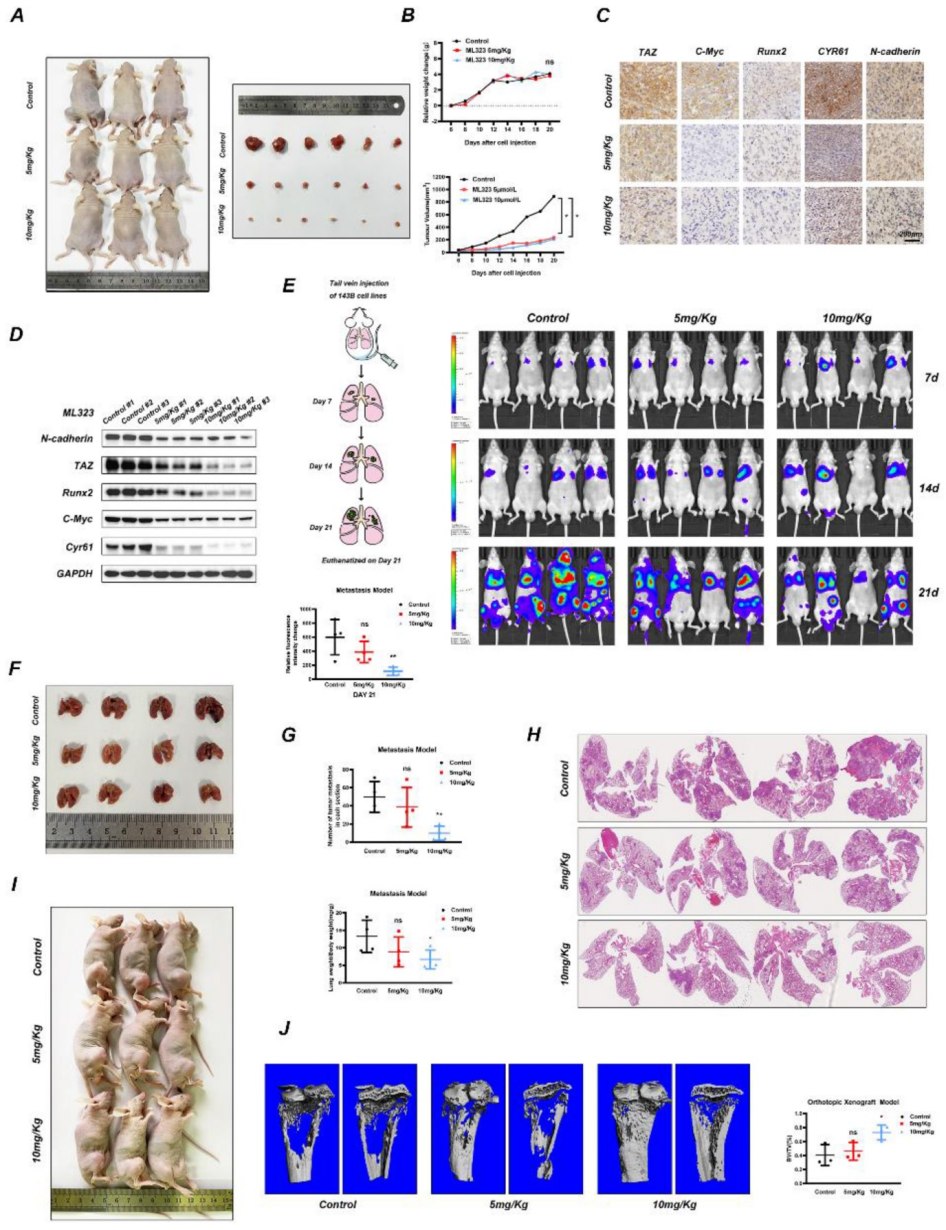
** USP1 inhibition by ML323 suppresses OS growth *in vivo*. A-D.** Subcutaneous xenograft tumorigenesis model was established and randomized to three groups for intraperitoneal injection of PBS (negative control) or ML323 (5 and 10 mg/Kg) every two days as indicated. The nude mice were sacrificed three weeks after intraperitoneal injection and the tumors were isolated from the nude mice and arrange neatly according to each groups (A). The tumor volume and weight of nude mice were measured every two days and arranged into line charts (B). Immunohistochemistry (IHC) and Western blot assay revealed the relative content of TAZ, C-Myc, Runx2, Cyr61 and N-cadherin in tumors from different groups (C and D). **E-H.** A tail vein metastasis model was established and treated as mentioned. Tumor metastasis of each nude mice were imaged by an *in vivo* bioluminescence imaging system on day7, day14 and day21, respectively (E). The nude mice were euthanatized on day21 and the lungs were harvested for photograph and H&E staining (F, G and H). **I, J.** An orthotopic xenograft model was established as mentioned and intraperitoneal administration with PBS (negative control) or ML323 (5 and 10 mg/Kg) every two days. Then the nude mice were euthanatized three weeks after intraperitoneal injection and the tibia was preserved for micro-CT scanning. Data represents the means ± SD. The images and data presented were acquired from and represented three independent experiments. P*< 0.05, P** < 0.01, P*** < 0.001 in comparison with the control group.

We then evaluated whether ML323 can exert inhibitory effects on OS progression *in vivo*. Accordingly, we established a subcutaneous xenograft tumorigenesis model and the treated nude mice were then randomly divided into three groups and subjected to intraperitoneal injection with vehicle solution (Control) or ML323 (5 or 10 mg/kg) every 2 days for 3 weeks. The results showed that ML323 suppressed tumor growth in an ML323-dose-dependent manner (Fig. 5A and 5B) and in the absence of fluctuations in the weights of mice from each group (Fig. 5B). We then evaluated the levels of tumor proteins from each group by immunohistochemical analysis, finding decreased levels of TAZ, C-MYC, RUNX2, N-cadherin, and CYR61 in ML323-treated tumors relative to those in controls (Fig. 5C), with these results confirmed by western blot analysis as well (Fig. 5D). To assess the toxicity of ML323 to non-tumor cells, we preserved several vital organs of nude mice from different groups and performed hematoxylin and eosin (H&E) staining, which revealed no apparent difference histology between groups (Fig. S4A). Moreover, a tail vein metastasis model was established to assess the anti-metastasis capacity of ML323 *in vivo*. Tumor metastasis of each nude mice were imaged by an *in vivo* bioluminescence imaging system on day7, day14 and day21, respectively. The results revealed that the relative fluorescence intensity changes were slowed with the treatment of ML323 in high dosage (10 mg/Kg) (Fig. 5E). Meanwhile, we found that systemic treatment of ML323 in high dosage by intraperitoneal injection significantly decreased the number of metastatic nodules in lungs (Fig. 5F, 5G and 5H). Another orthotopic xenograft model revealed similar results. Administration of ML323 in high dosage significantly suppressed growth and bone destruction of OS cells *in situ* (Fig. 5I and 5J). Collectively, the above results demonstrated that USP1 inhibition by ML323 suppressed the progression of OS *in vivo* and in the absence of significant cytotoxicity.

## Discussion

Imbalance between the ubiquitination and deubiquitination of proteins profoundly affects tumor growth and invasion. As key regulators in the process of deubiquitination, DUBs play important roles in tumor-based diseases and represent potential diagnostic and therapeutic targets in the treatment of cancer 9. USP1 is among the best-characterized human DUBs and implicated in multiple segments of the DNA-damage-repair pathway, including regulation of FANCD2 and PCNA by deubiquitination 13-15. Meanwhile, USP1 is reportedly a potential target in regulating the progression of breast cancer 18, 20, non-small-cell lung cancer (NSCLC) 21, 22, and hepatocellular carcinoma 23. Additionally, USP1 maintains stem cell properties in OS by deubiquitinating IDs 16. Silencing USP1 in U2OS cells (a human OS cell line) attenuated U2OS proliferation and invasion 17. However, its specific role and underlying mechanisms in OS progression remain poorly understood.

Based on Ashley Mussell et al, USP1 and TAZ form a complex that alters TAZ ubiquitination and relates to the metastatic properties of breast cancer 20. Consistent with their findings, our Co-IP assay, Silver staining, and immunofluorescence assay demonstrated direct binding between USP1 and TAZ in OS cell lines. As a key regulator of the Hippo signaling pathway, TAZ is ubiquitously expressed in bone marrow and involved in regulating bone metabolism 24, 25. Moreover, TAZ can initiate OS progression and metastasis 26, resulting in poor prognosis and increased chemoresistance 27. TAZ was also proved to be phosphorylated by the upstream kinases LATS1/2, resulting in ubiquitination and proteasomal degradation 26. Our findings revealed that the expression of USP1 and TAZ was consistent elevated in OS tissues and cell lines. Consequently, we assume that TAZ may act as a substrate of USP1 that partially accounts for the oncogenic role of USP1 in OS. Interestingly enough, our results presented that USP1 deletion by SiRNA accelerating the degradation of TAZ while with no obvious effects on its mRNA expression. MG132 rescue assays along with Co-IP assays further demonstrated that USP1 deletion promotes TAZ poly-ubiquitylation via K11 and K29 linkage for subsequent proteasomal degradation in OS cells. Additionally, as a co-activator, TAZ functions by translocating into the nucleus, where its role in EMT and cell growth is mediated by interacting with TEAD transcription factors 13, 28, 29. TAZ in the nucleus can also initiate RUNX2, Wnt and β‐catenin activities 26. Of which, RUNX2 was reported to be involved in the metastasis and chemoresistance of OS 30. Targeting the Wnt/β-catenin pathway can also exert inhibitory effects on EMT phenotype and distant pulmonary metastasis in OS 31. Our current results presented that USP1 deletion reduced TAZ accumulation in the nucleus along with TAZ interactions with TEAD transcription factors, and decreased the expression of downstream genes (CYR61, c-Myc, and RUNX2) involved in Hippo signaling pathway. In addition, overexpression of TAZ in OS cell lines partially abolished the anti-proliferation effects of USP1 deletion. Whereas, the effects of TAZ overexpression on the USP1-mediated migration capacities varied within different cell lines. As presented in Fig. 3C, USP1 deletion slowed the wound healing in osteosarcoma cells, while overexpression of TAZ merely reversed this performance in HOS cells. Besides, transwell assays exhibited that TAZ has no significant rescue effects on the invasive capacity of USP1. Altogether, the above results indicated that the regulatory role of USP1 in OS can be partially achieved by post-translational modification of TAZ. Nevertheless, given to the slight differential performance of TAZ overexpression in 143B and HOS cell lines, the rescue role of TAZ in USP1 remains to be further explored. This is also one of the limitations of our study. Besides, the mechanisms by which USP1 regulates osteosarcoma cell invasion is also what need to be addressed in the future.

Chemotherapy plays a critical role in OS treatment. Although the prognosis of OS patients has improved significantly since introduction of neoadjuvant chemotherapy in the 1970s 33, 34, increased incidence of chemoresistance has resulted in the decreased efficacy of chemotherapy in some OS patients 35. Moreover, the adverse effects of chemotherapy on normal cells can also limit its clinical application 36. Given that ML323, a selective inhibitor of USP1, presented the abilities to sensitize resistant OS cells to cisplatin 19 along with anti-metastatic effects in breast cancer 18. Further exploring the role of ML323 in osteosarcoma may address the current therapeutic limitations and reveal new strategy for the clinical treatment of OS. Thus, in the present study, we used ML323 to pharmacological interfere with USP1 activity in order to explore its specific role in OS. Our results showed that USP1 inhibition by ML323 had the similar impact on Hippo signaling pathway and effectively suppressed the migration, proliferation, EMT, and metastasis of OS both *in vitro* and *in vivo*. Notably, as a selective inhibitor of USP1, ML323 exerts its inhibitory effect through an allosteric mechanism rather than reducing USP1 expression 32. Indeed, western blot revealed that USP1 expression was upregulated by ML323 treatment, which could be partially explained by negative feedback.

In conclusion, our research identified a novel molecular mechanism underlying the function of USP1 in OS. Briefly, USP1 regulates the ubiquitylation of TAZ via K11 and K29 chains and influences the downstream of Hippo signaling pathway, which partially accounts for its regulatory role in OS. In addition, our findings also suggest ML323 as a potentially promising therapeutic approach to OS treatment.

## Supplementary Material

Supplementary figures.

## Figures and Tables

**Figure 6:**
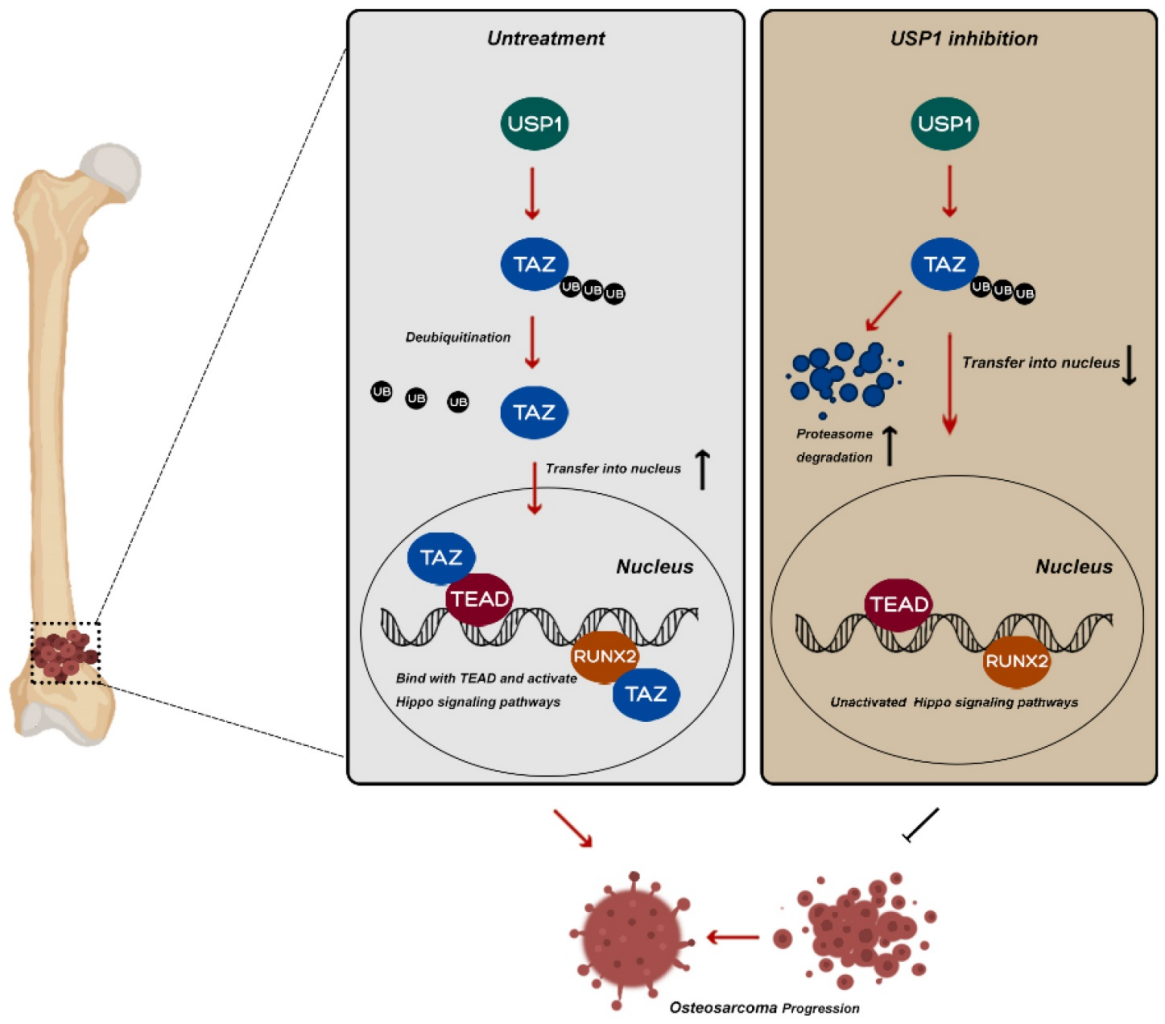
** Potential molecular mechanism involved in USP1 inhibition in osteosarcoma.** USP1 depletion or ML323 treatment suppressed the progression of osteosarcoma via destabilizing TAZ and regulating Hippo signaling pathway.

**Table 1:** The sequences of primers utilized in this experiment

Gene	Primers	
β-Actin	Forward: 5'-GATGAGATTGGCATGGCTTT-3'	Reverse: 5'-CACCTTCACCGTTCCAGTTT-3'
E-cadherin	Forward: 5'-CTCGACACCCGATTCAAAGT-3'	Reverse: 5'-GCGTGACTTTGGTGGAAAAC-3'
N-cadherin	Forward: 5'-AGGATCAACCCCATACACCA-3'	Reverse: 5'-TGGTTTGACCACGGTGACTA-3'
Vimentin	Forward: 5'-AGTCCACTGAGTACCGGAGAC-3'	Reverse: 5'-CATTTCACGCATCTGGCGTTC-3'
MMP13	Forward: 5'-ACTGAGAGGCTCCGAGAAATG-3'	Reverse: 5'-GAACCCCGCATCTTGGCTT-3'
TAZ	Forward: 5'-CACCGTGTCCAATCACCAGTC-3'	Reverse: 5'-TCCAACGCATCAACTTCAGGT-3'
