# Supplementary material for: USP1 inhibition suppresses the progression of osteosarcoma via destabilizing TAZ
Source: Int J Biol Sci. 2022 May 1;18(8):3122–36. doi: 10.7150/ijbs.65428 (PMC9134912; doi:10.7150/ijbs.65428)
Supplement: Supplementary file 1 — Supplementary figures. [file ijbsv18p3122s1.pdf]

Supplementary Figures and Legends

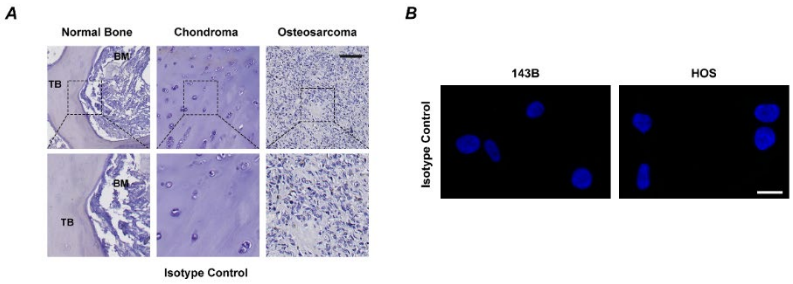

Supplementary Figure 1. Isotype control staining of the tissues and cells.

A．Isotype control IHC of normal bone, chondroma and osteosarcoma sections. Scale bar=100μm. **B.** Isotype control IF of 143B and HOS cell lines. Scale bar=20μm.

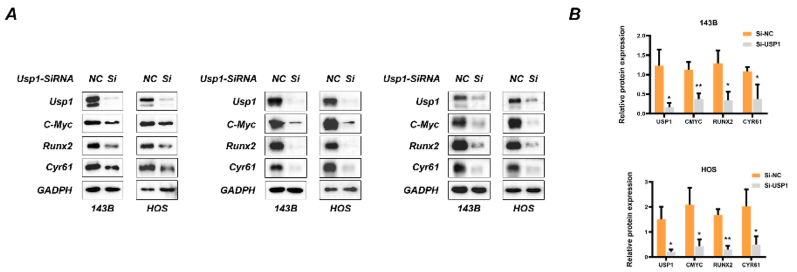

Supplementary Figure 2 USP1 deletion reduces the expression of downstream genes in Hippo signaling pathway.

A. The expression of Usp1, C-MYC, Runx2 and Cyr61 was determined by western blot

assay under the condition of USP1 deletion. **B.** The quantification of relative protein expression.

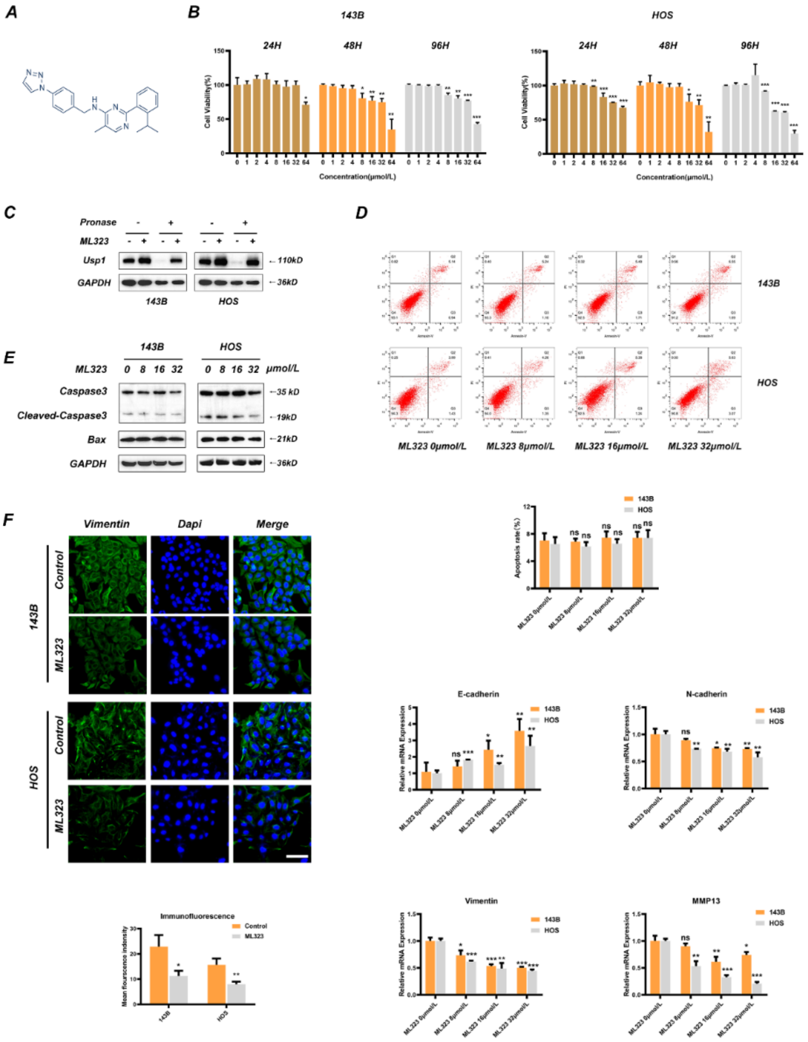

Supplementary Figure 3. ML323 suppresses EMT phenotype of OS cell lines while with no obvious effect on apoptosis.

A. Molecular formula of ML323. **B.** 143B and HOS cells were incubated with different concentrations of ML323 for 24/48/96h, and the cell viability were analyzed by CCK-8 assays. **C.** DARTS assays were performed to identify the interaction between USP1 and ML323. USP1 was resistant to the degradation effect of pronase in case of ML323

treatment, which identified USP1 as the pharmacological target of the ML323. **D. E.** USP1 inhibition by ML323 exerted no obvious effects on the apoptotic levels of OS cells. Proteins involved in apoptosis (Caspase3, Cleaved-Caspase3 and BAX) were detected in OS cells by Western blot assay after the treatment of ML323 as indicated for 48 (D). OS cells with different treatment as indicated were stained by PI and V-FITC, then analyzed through flow cytometry (E). **D.** As determined by Immunofluorescence assay, the expression of Vimentin in OS cells was decreased in the presence of ML323 by comparing with the control group. **E.** The expression levels of EMT phenotype-related genes were measured by qRT-PCR after the stimulation with different dosage of ML323 range from 0 to 32 μmol/L as indicated for 48h. Data represents the means ± SD. The images and data presented were acquired from and represented three independent experiments. $P^* < 0.05$, $P^{**} < 0.01$, $P^{***} < 0.001$ in comparison with the control group.

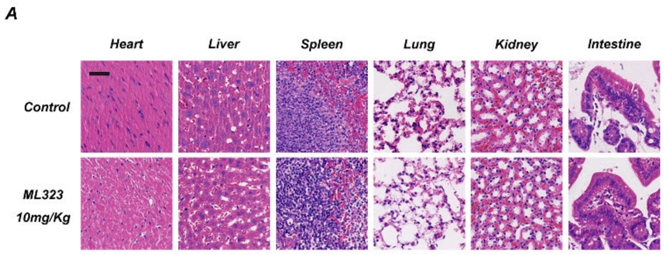

Supplementary Figure 4. ML323 has no obvious toxic effect on nude mice

A. Vital Organs from Control and ML323(10 mg/kg) groups were preserved for H&E staining.
